# Supplementary material for: Predicting real-world navigation performance from a virtual navigation task in older adults
Source: PLoS One. 2025 Jan 27;20(1):e0317026. doi: 10.1371/journal.pone.0317026 (PMC11771902; doi:10.1371/journal.pone.0317026)
Supplement: Attachment — Submitted filename: Response_Predicting_real-world_navigation_in_older_adults_auresp_2.docx [file pone.0317026.s002.docx]

We are grateful to the reviewers for their feedback. We have revised the manuscript in light of these recommendations. Below, we outline how we have addressed the comments by the reviewers. We include the original review comments in black font, our reply in blue font, and quotes from our manuscript in red font. Overall, we have made the following revisions:

Reviewer #2: Thank you for providing a detailed explanation for the reviewer comments. I feel this manuscript has improved a lot. However, there was one comment of my previous review that was not addressed. I feel that this Methods – Statistical analysis section needs to be rewritten profoundly before accepting this paper. I would like to see an extensive statistical paragraph where the research questions and how these will be analyzed are written in detail. For full transparency, I have added my previous feedback below:

Methods – statistical analysis: I miss how you will analyze your research question, namely whether Sea Hero Quest is predictive of real-world navigation ability in an older population. As you are also expecting to find a general decline reflected in a difference between the older group and the younger group of your previous study, this also has to be described in the statistical analysis part how you will test this. If you are including testing for differences on the basis of gender, this also has to be included in the aims at the end of the introduction.

Thanks a lot for bringing this up. It is true that the Statistical analysis section needed to be rewritten. It now reads as follows:

To test whether Sea Hero Quest is predictive of real-world navigation ability, we calculated Pearson’s correlations between real-world wayfinding performance and the virtual performance at each SHQ wayfinding level.

To assess the cognitive decline in the real and the virtual environments, we compared the performance of older versus younger participants. In both environments, we used a chi-square test to compare the proportions of participants in the older versus younger cohorts who completed the task before the time limit.

We also examined whether familiarity with the video game controls might play a role in driving navigation performance. To explore this, we examined whether the training level (Level 1) was correlated with real-world performance for our older group. Since Level 1 is not an assessment of spatial navigation ability but rather video game familiarity / visuo-motor skill (the goal remains visible from the start of the Level), we used Pearson’s correlation analysis to test whether there was a correlation between the distance travelled in Level 1 and the normalised distance in the real world.
