# Supplementary material for: Predicting real-world navigation performance from a virtual navigation task in older adults
Source: PLoS One. 2025 Jan 27;20(1):e0317026. doi: 10.1371/journal.pone.0317026 (PMC11771902; doi:10.1371/journal.pone.0317026)
Supplement: Attachment — Submitted filename: Response Predicting ... older adults 131224.docx [file pone.0317026.s003.docx]

Dear editor, dear reviewers,

Thanks a lot for this further round of review. There was only the following outstanding comment:

The paper has improved after this second review but still one aspect was not addressed. A reference for the Navigational Strategy Questionnaire (NSQ) is still missing. If the following reference was the one used, please include it, otherwise include the correct one.

Zhong, J. Y., & Kozhevnikov, M. (2016). Relating allocentric and egocentric survey-based representations to the self-reported use of a navigation strategy of egocentric spatial updating. Journal of Environmental Psychology, 46, 154-175

We have now included the reference in question for the NSQ into the manuscript. Thanks so much for pointing this out, and thank you for all of the editorial and reviewing work.
